# Divergent and convergent evolution in metastases suggest treatment strategies based on specific metastatic sites

**DOI:** 10.1093/emph/eov006

**Published:** 2015-03-20

**Authors:** Jessica J. Cunningham, Joel S. Brown, Thomas L. Vincent, Robert A. Gatenby

**Affiliations:** ^1^Cancer Biology and Evolution Program, Moffitt Cancer Center, Tampa, FL 33612; ^2^Department of Biological Sciences, University of Illinois at Chicago, Chicago, IL 60607; ^3^Aerospace and Mechanical Engineering, University of Arizona, Tucson, AZ, 85745, USA

**Keywords:** metastases, evolution, cancer therapy, evolutionary game theory

## Abstract

**Background and objective:** Systemic therapy for metastatic cancer is currently determined exclusively by the site of tumor origin. Yet, there is increasing evidence that the molecular characteristics of metastases significantly differ from the primary tumor. We define the evolutionary dynamics of metastases that govern this molecular divergence and examine their potential contribution to variations in response to targeted therapies.

**Methodology:** Darwinian interactions of transformed cells with the tissue microenvironments at primary and metastatic sites are analyzed using evolutionary game theory. Computational models simulate responses to targeted therapies in different organs within the same patient.

**Results:** Tumor cells, although maximally fit at their primary site, typically have lower fitness on the adaptive landscapes offered by the metastatic sites due to organ-specific variations in mesenchymal properties and signaling pathways. Clinically evident metastases usually exhibit time-dependent divergence from the phenotypic mean of the primary population as the tumor cells evolve and adapt to their new circumstances. In contrast, tumors from different primary sites evolving on identical metastatic adaptive landscapes exhibit phenotypic convergence. Thus, metastases in the liver from different primary tumors and even in different hosts will evolve toward similar adaptive phenotypes. The combination of evolutionary divergence from the primary cancer phenotype and convergence towards similar adaptive strategies in the same tissue cause significant variations in treatment responses particularly for highly targeted therapies.

**Conclusion and implications:** The results suggest that optimal therapies for disseminated cancer must take into account the site(s) of metastatic growth as well as the primary organ.

## Abstract (precis)

Cancer cells, although maximally fit at their primary site, typically have lower fitness on the adaptive landscapes offered by the metastatic sites due to organ-specific variations in mesenchymal properties and signaling pathways. Clinically evident metastases will exhibit time-dependent divergence from the phenotypic mean of the primary population as the tumor cells evolve and adapt to their new circumstances. In contrast, tumors from different primary sites evolving on identical metastatic adaptive landscapes exhibit phenotypic convergence so that, for example, metastases in the liver from different primary tumors will evolve toward similar adaptive phenotypes. The combination of evolutionary divergence from the primary cancer phenotype and convergence towards similar adaptive strategies in the same tissue cause significant variations in treatment responses particularly for highly targeted therapies.

This suggest that optimal therapies for disseminated cancer must take into account the site(s) of metastatic growth as well as the primary organ.

## INTRODUCTION

Currently, primary tumors can usually be controlled or eradicated leaving metastases as the cause of death in ∼90% of cancer patients [1–6]. The metastatic cascade includes invasion of tumor cells into lymphatic or blood vessels at the primary site, circulation, arrest in a distant organ, extravasation into the adjacent tissue and proliferation to form a new invasive (metastatic) cancer [2, 3]. Development of metastases is extremely inefficient [7–11]. In experimental systems, e.g. over 80% of circulating tumor cells (CTCs) survived that vascular transit and impacted at a distant site but only 2% of CTCs produced micro-metastases and only 1% of these micro-metastases (0.02% of cells injected) progressed to macroscopic tumors [5]. Similarly, CTCs are present in numerous cancer patients who do not manifest a metastatic disease. In fact, primary tumors can shed millions of cells into circulation per day without apparent metastatic tumor formation [12]. Thus, while the metastatic process requires multiple steps, the clinical outcome, once tumor cells enter the circulatory system, is largely dependent on the interactions between these cells and the microenvironment of a distant organ site. Unfortunately, although formation of clinical metastases from CTCs is rare, the sheer magnitude of CTCs makes metastatic disease likely given enough time.

We propose that the formation of metastases from CTCs is a Darwinian process involving evolution of the tumor cells’ phenotype along the new adaptive landscape provided by the novel tissue environment. Only tumor cells that are already sufficiently adapted or that can evolve adaptations to local growth constraints in the ‘foreign’ landscape of the distant organ can form metastases. These evolutionary dynamics result in phenotypic divergence of the metastatic cells away from their source population at the primary site, but evolutionary convergence of metastatic cells when colonizing the same organ.

Specific therapies for metastatic disease are currently dictated exclusively by the site of the primary tumor. However, our results demonstrating evolutionary divergence of metastatic populations from the primary tumor and convergence of metastases towards optimal strategies for the specific metastatic site, suggest that this approach will not necessarily be best. Although ‘mixed response’ (site-to-site variations in response in the same patient) is well known in clinical oncology, it has not been extensively studied. Recently, quantitative imaging techniques have demonstrated mixed responses to therapy in 25–50% of cases [13–19]. Here, we present computer simulations of targeted therapy that suggest treatment strategies for patients with disseminated cancers should be based both on the organ of the primary tumor and the recipient organ site(s) of the metastases.

## METHODOLOGY

### Evolutionary game theory in multicellular tissue

We describe the interactions of tumor and normal cells with each other and with the microenvironment using evolutionary game theory [20–22]. Evolution by natural selection is modeled as a game with players, strategies, strategy sets and payoffs. The players are the individual cells. The strategies are heritable phenotypes available to the individual players. A cell’s strategy set is determined by all evolvable strategies that could occur via mutations and/or epigenetic changes within a biologically feasible time period. This approach explicitly links cellular proliferation to its ‘fitness’, which is defined as the per capita growth rate of cells possessing a particular strategy. Fitness emerges from a cell’s given strategy, the strategies and densities of other neighboring cells, and the other properties of the local microenvironment. In this evolutionary game, winners proliferate at the expense of losers so that phenotypic properties are retained or lost depending on their contribution to fitness.

The *in vivo* evolutionary dynamics are visualized through adaptive landscapes. The adaptive landscape plots a tumor cell’s fitness as a function of its strategy. It provides a geometric illustration of a tumor cell’s evolutionary potential as a result of its strategy, its interactions with all extant cell populations including products of their phenotypes such as growth factors and the extracellular matrix. The adaptive landscape is a plot of the fitness of any phenotype in some microenvironment compared with all potential cellular phenotypic strategies both extant and available through somatic evolution.

Tumor cell populations evolve through time-dependent changes in their mean phenotype. The direction of change is ‘up’ the slope of the adaptive landscape, and the rate of change is proportional to the magnitude of this slope. Additionally, the rate of evolution increases with increases in phenotypic variance produced by mutations or epigenetic changes. Through natural selection, phenotypes conferring higher survival and proliferation replace less successful strategies via clonal expansion.

Thus, normal cells, with a low background mutation rate, will exhibit little to no evolutionary change. Whereas, tumor cells with a high background mutation rate and/or exposure to non-physiological, toxic environments, such as inflammation or hypoxia, evolve and adapt much more rapidly.

The shape of the landscape depends on the phenotypic properties of the extant cell populations and the number of individuals within each population. The landscape changes over time as changes in cell density and cell phenotype frequencies alter the environment, and these microenvironment changes, in turn, alter phenotype-specific fitness. The ecological and evolutionary dynamics of cancer growth and invasion are governed by these interdependent processes. Environmental circumstances favor specific adaptations which, in turn, alter the environment creating new selection pressures, driving further phenotypic evolution and so on.

### The mathematical model

Evolutionary game theory is applied to cancer by assuming *n*_s_ cellular phenotypes within some given tissue volume. These include the extant clonal populations as well as all possible distinct phenotypes to which those populations can evolve through temporary or permanent alterations in gene expression. Each population consists of $x_{i}$ individuals using strategies $u_{i},\;i=1,\;\ldots ,\;n_{\text{s}}$. We define a population vector x, and mean phenotype strategy vector u:

$$x=[x_{1}\;\ldots \;x_{i}]$$

$$u=[u_{1}\;\ldots \;u_{i}]$$

Each phenotype represents a unique pattern of observable traits that affect proliferation. For example, a trait could represent one of the ‘hallmarks’ of cancer [23]. Then, distinct populations are defined by the value assigned to each hallmark. Thus, a phenotype that is fully responsive to growth inhibitors might be assigned a value of 0 while a phenotype that is totally unresponsive to all inhibitory growth signals is assigned a value of 1 with intermediate sensitivity assigned values between 0 and 1.

Note that the values assigned to each trait are dependent on both the properties of the cells and the properties of other populations and their phenotypes. For example, normal liver cells respond to a different set of growth promoters and inhibitors than normal colon cells. A tumor population developing in the colon will evolve adaptations to growth promoters and inhibitors in the colonic mucosa. However, these cells will likely not respond identically to growth factors in other organs since these do not constitute selection forces in the primary site of somatic evolution.

Although each population possesses a mean phenotype, the model assumes some phenotypic diversity around this mean caused by random mutations, epigenetic changes or environmental variations such as hypoxia and acidosis within the premalignant lesion. This heritable variance *σ* is a necessary condition for evolution.

In each somatic ecosystem, we define the fitness generating function of an individual using strategy v in a tumor where u and x are the extant phenotypes and their population sizes, respectively, within the tumor. By replacing v with one of the strategies present in the population, say $u_{i}$, one obtains a function that gives the per capita growth rate of the clonal lineage with this strategy as a function of the other strategies and their population densities within the tumor. Each organ offers different fitness opportunities and hazards to a tumor cell with a particular strategy. Hence, each organ can be represented by a different G-function.

With this definition, the population dynamics of any clonal lineage, $u_{i}$, can be written as:

(1) $$\frac{\partial x_{i}}{\partial t}=\;x_{i}G{\left(v,\;u,\;x\right)}_{v=u_{i}}$$

The G-function also provides the adaptive landscape as the plot of G versus v, for fixed u, x.

By describing the cellular phenotype through distribution functions, the models allow population evolution as some individuals in the distribution curve proliferate more than others causing the mean phenotype of a tumor cell lineage to change with time.

With heritable phenotypic variance σ, the rate of evolutionary change of $u_{i}$ is

(2) $$\frac{\partial u_{i}}{\partial t}=\sigma_{i}^{2}{\frac{\partial G}{\partial v}|}_{v=u_{i}}$$

Note that ∂G/∂v is the local slope of the adaptive landscape and it represents the direction and magnitude of selection pressures acting on the clonal lineage in its environment. The heritable variation, *σ*, is solely dependent on intracellular factors such as the mutation rate.

In the simulations below we assume that the background mutation rate in normal cells is sufficiently small that they cannot evolve during the time scales under consideration (i.e. σ≈0 for all normal cells).

### Evolutionary model of primary and metastatic cancers

We write the cell dynamics of normal and malignant tissue as a Lotka-Volterra competition model in which epithelial cells (whether normal or malignant) interact with the mesenchymal populations:

(3) $$G\left(v,\;u,\;x\right)=r\left(1-\;\frac{1}{K\left(v\right)}\text{n}\underset{j}{\sum }a_{j}\left(v,\;u_{j}\right)\text{n}x_{j}\right)$$

where r is the cellular intrinsic growth rate, K(v) is the total carrying capacity of the environment for all (epithelial and mesenchymal) cells, and $a_{j}(v,\;u)$ is the term that specifies the interactions between cell populations. We propose that carrying capacity (K) is dependent on blood flow which can depend on the cancer cell’s strategy (depending on a number of factors such as production of angiogenic factors). This interaction between carrying capacity and the angiogenic strategy of the tumor cells is expressed by the following equation.

(4) $$K\left(v\right)=\;K_{max}\text{*exp}\left(-\frac{v^{2}}{2\sigma_{K}^{2}}\right)$$

Under this formulation, carrying capacity declines according to a Gaussian curve as a cell’s strategy, v, deviates from 0. At v=0, the carrying capacity is maximized at *K*_max_. The rate of decline in carrying capacity as the cell’s strategy deviates from 0 is determined by the variance term of the Gaussian curve, $\sigma_{\text{K}}^{2}$. The tumor cell’s strategy shall represent an evolutionary trade-off in which the tumor cell cannot simultaneously maximize its carrying capacity and minimize its competitive interactions with other cells including the mesenchyme as outlined below.

For the competition term, *a*, of the Lotka-Volterra competition model we assume that individuals sharing the same phenotype compete most intensely (like interacts most with like) with a caveat that competition is asymmetric. Cells with a larger strategy value (more ‘aggressive’) have a larger negative effect on cells with a smaller strategy value than vice versa. We will let competition be most extreme on a cell with strategy v when the competing clonal lineage has a strategy of $u_{j}=v+\beta$ for β>0. Like the carrying capacity function in Equation (4), we will use a modified Gaussian function to describe the competitive effect of individuals with strategy $u_{j}$ on a focal call with strategy, v. We let a variance term $\sigma_{a}^{2}$ determine how quickly the competition coefficient changes as competitors deviate in their strategy values.

(5) $$a\left(v,\;u_{j}\right)=1+\exp\left(-\frac{{\left(v-u_{j}+\beta \right)}^{2}}{2\sigma_{a}^{2}}\right)-\exp\left(-\frac{\beta^{2}}{2\sigma_{a}^{2}}\right)$$

This function has been scaled so that two epithelial cells (normal or malignant) will have a competition coefficient of a=1 if they share the same strategy, and this competition term takes on a maximum value when $u_{j}=v+\beta$.

The evolving strategy in this model, v, can be thought of as some overall resource acquisition and interaction strategy used by the epithelial cells when interacting with local mesenchymal cells. This strategy trades off maximally inducing mesenchymal cells to form vasculature versus successfully competing with other cells including the mesenchymal cells.

A critical factor for the malignant tumor cells as they evolve to a fitness peak in the adaptive landscape involves modulating their interactions with normal mesenchyme. In our model, the mesenchymal cells do not evolve but they do have significant amount of phenotypic plasticity that strongly contributes to the robustness of normal tissue (allowing, e.g. healing after infection or injury by forming new vessels and scar tissue). We suppose that the interactions between cancer cells and mesenchymal cells at the primary site, and more importantly at the secondary metastatic sites, have two dominant features. First, tumor cells maintain and increase their fitness (proliferation) by inducing the mesenchymal cells to form blood vessels, produce extracellular matrix, and secrete local growth factors. Tumor cell fitness requires that it promote mesenchymal proliferation and function. Second, mesenchymal cells can proliferate extensively (forming scars e.g.) and by doing so can, in effect, compete with cancer cells for space and substrate. In fact, a well-known limitation of primary cell cultures is the propensity of mesenchymal cells to overgrow the dish and kill the tumor cells. We suppose this tradeoff dominates the tissue specific selection pressures for tumor evolution.

Thus the value assigned to v reflects variations in the cancer cell strategy for resource allocation. Cells with a strategy value of 0 maximize their carrying capacity by promoting mesenchymal cells that enhance and maintain blood flow. At the other extreme, tumor cells with higher strategy values sacrifice this carrying capacity but may enhance their proliferation by suppressing the growth of mesenchymal cells which also compete for space and substrate.

## RESULTS

### Normal tissue adaptive landscape

**Figure 1.**
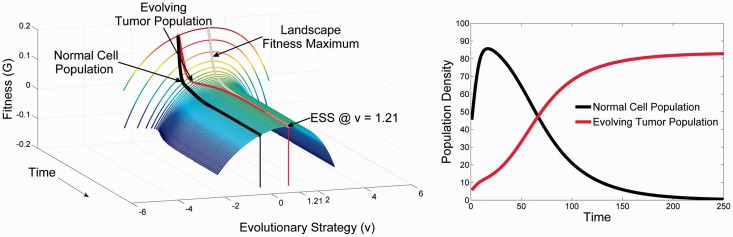
The temporal dynamics (Time) of the adaptive landscape (Fitness, *G*) versus the Evolutionary strategy of a focal cell (*v*) **Panel A**: At time 0, the normal cell population possesses a strategy value (normalized to *u* = 0, where *u* is the phenotype of the extant population of cells) and an equilibrium cell density (shown in **Panel B**) that is neither evolutionarily nor ecologically stable in the face of a cancerous cell. Normal whole organism homeostatic processes means that the normal cells’ phenotype is not at a fitness peak and their fitness (G) would be greater than 0 if cell proliferation were not regulated. At the homeostatic equilibrium shown at time 0, we introduce a rare cancerous cell line that has mutated from a normal cell. **Panel A** shows the temporal changes to the adaptive landscape (smooth surface) and the evolutionary divergence of the tumor cell’s phenotype (red line) from the normal cell’s (black line) as it evolves up the slope of the adaptive landscape. All the while the tumor cell’s population density is growing (red line in **Panel B**) at the expense of the normal cells (black line), this increase in population size forces the adaptive landscape down, until the tumor cells have zero fitness and *G* = 0 and the normal cells have negative fitness of *G* = −0.024. At the ESS, the tumor cell’s strategy of *u* = 1.2 balances its competitive ability with its carrying capacity. The end result is a malignant tumor that outcompetes the normal epithelial cells and co-opts the mesenchyme

Figure 1 demonstrates the typical adaptive landscape of normal epithelial and mesenchymal cells. We assume phenotypic stability with time (i.e. no evolution is permitted) and the following parameter values r = 0.25, $K_{\text{max}}$ = 100, $\sigma_{\text{K}}^{2}=\sigma_{a}^{2}=4$ and β=2.

The normal cells possess a strategy of u=0, as in our model, this is the most efficient one for the organ and the whole organisms. Notice that the normal cell populations do not occupy a fitness maximum of the adaptive landscape, nor does their equilibrium population size achieve its carrying capacity. Here, normal tissue is maintained homeostatically at a tissue density well below what unlimited growth would permit. Thus, the population density is x=40, less than the true carrying capacity of the environment, *K*_max_ = 100. This translates well into a model of cancer biology. Normal epithelial surfaces typically consist of a layer of epithelial cells bound to a basement membrane with a large adjacent open space (such as in a duct, bronchus or the surface of the colon). The ecological robustness of the normal tissue landscape can be analysed by examining the system following perturbations in cell population size. We find that the adaptive landscape of normal tissue in general possesses ecological stability. That is, following alterations in the number of individuals within extant populations by, e.g. injury, inflammation or infection, the system will return to its steady state over time. Clearly this is a property necessary for maintaining tissue integrity despite a wide range of environmental stresses and injures. In contrast, the system is not evolutionarily stable, meaning the system will not necessarily return to baseline if a new phenotype arises in or enters the landscape.

Although the normal tissue adaptive landscape gives rise to stability for normal cell populations, it also permits an opportunity for the growth of cancerous mutant populations. That is, if a cellular population residing at one of the fitness maxima should emerge due to mutations, it will stably dominate the landscape, driving other phenotypes to extinction forming an invasive cancer. Thus, while the strategy of the normal tissue is well configured to form robust functioning multicellular tissue, its strategy and position on the adaptive landscape renders it vulnerable to tumor formation. In other words, the potential for cancer development and growth is a ‘cost’ or ‘penalty’ for multicellular organisms in maintaining tissues and organs that serve the whole organism.

### Primary cancer adaptive landscapes

We assume the dynamics that give rise to a primary carcinoma arise from unrestrained proliferation that permits primary tumor cell to grow to their carrying capacity, and the ability of these cancerous epithelial cells to evolve with time. Evolution is possible for two reasons: (i) the transformed epithelial cells ‘can’ evolve because they undergo random genetic and epigenetic events that produce heritable phenotypic changes. (ii) The adaptive landscape presented by normal tissue ‘allows’ evolution because the extant cellular populations do not occupy a fitness maximum (resulting in an ‘unrealized evolutionary opportunity’) or fill all available space (resulting in an unrealized ‘ecological opportunity’). Thus, a cellular population that can evolve has an opportunity to proliferate into available unoccupied space such as the lumen of a duct. The opportunity to both acquire new heritable properties and proliferate in response to these properties allows tumor populations to move up the adaptive landscape and arrive at the vacant fitness peak. Interestingly, even as the strategies of the tumor populations evolve up the adaptive landscape, the proliferation of these cells towards their carrying capacities pushes the adaptive landscape down as available space and nutrient opportunities are filled.

The adaptive landscape of a primary cancer is shown in Fig. 1. Note that the configuration of the normal tissue has been drastically changed. The tumor population, once near equilibrium (zero fitness), now occupies a fitness peak forming an evolutionarily stable state (ESS). By reaching a maximum on the adaptive landscape the tumor cells are evolutionarily stable, and by growing to their carrying capacity they are ecologically stable by depressing their fitness to zero net population growth.

### Evolutionary dynamics of metastases

**Figure 2.**
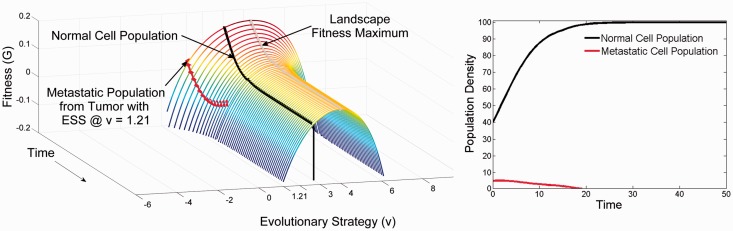
Failure to metastasize when the recipient organ offers a substantially different environment and adaptive landscape than the organ of origin. **Panel A** at time 0 shows the normal cells at their homeostatic equilibrium and with a strategy of *u* = 3; a strategy that is appropriate to this organ (it is the strategy that maximizes the carrying capacity of the organ). As normal cells they possess a restrained strategy relative to the adaptive landscape (a higher strategy value could invade) and they are regulated at a cell density below their carrying capacity (40 instead of 100). At time 0, a small number of malignant tumor cells from the organ depicted in Fig. 1 invade. They arrive possessing the optimal strategy for the primary tumor, *u* = 1.2. However, this places them at a disadvantage on the adaptive landscape of recipient organ. They not only are far from what would be evolutionarily stable for the secondary site (*u* = 4.2), but because of competition from the normal cells they have negative fitness. Even as they evolve up the landscape (red line) their population declines to extinction (red line in **Panel B**). Extinction occurs before the tumor cells can evolve a strategy that would yield positive fitness. In the meantime the normal cells maintain their homeostatic equilibrium with a minor perturbation of their cell density (black line in **Panel B**) caused by the transient dynamic of the failed metastasis. This is likely fate of most metastases

Figure 2 represents the adaptive dynamics of a CTC impacting on a metastatic site that is very different from the primary tissue. Although very fit in its original primary carcinoma environment, is not fit in the secondary environment. The strategy used in competition with the epithelial and mesenchymal cells at the primary site is not necessarily a successful strategy at the new secondary site, especially as mesenchymal cells function in organ specific ways [24–29]. This configuration results in a negative fitness value (Fig. 2) which means the cancer cells cannot proliferate and will inevitably die off. Therefore, the introduction of invasive tumor cells will result in no clinical metastasis and no need for any type of treatment. This appears to be the most common fate of circulating cancer cells.

At the other extreme, CTCs may arrive at a tissue offering an adaptive landscape nearly identical to the primary site resulting in rapid, virtually immediate tumor growth. This outcome may seem intuitively unlikely. Nevertheless, with millions and even billions of CTCs encountering diverse tissue types, this scenario may explain the high frequency of certain metastatic diseases based on particular pairings of source and recipient organs. Interestingly, intravascular tumor cells may simply circulate back to the original source tissue where their ecological and evolutionary potential will be high. This outcome has been observed as ‘self-seeding’ [30].

**Figure 3.**
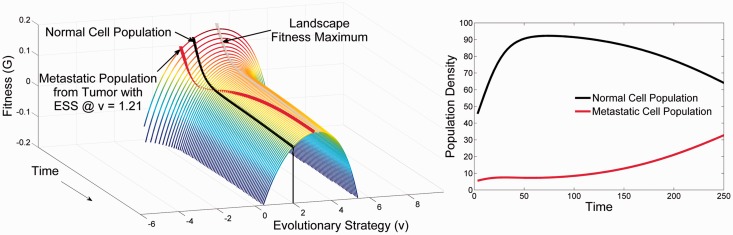
The evolutionary (**Panel A**) and ecological dynamics (**Panel B**) of a successful metastasis by malignant tumor cells into a secondary site that offers similar conditions to the primary tumor. The secondary site offers an adaptive landscape similar to the primary site. At time 0 the normal cells of this organ are at a homeostatic equilibrium of *u* = 2 and *x* = 40; *u* = 2 maximizes carrying capacity for this organ but the normal cells are regulated to a much lower cell density. Even though the malignant tumor cells arrive with a strategy of *v* = 1.2 (optimal for the primary tumor shown in Fig. 1), the low density of normal cells means that the malignant cells still possess positive fitness and can grow in density. This gives the cancer cells time to evolve up the adaptive landscape. Initially they converge on the strategy of the normal cells of this novel organ but then continue evolving the more aggressive phenotype of u = 3.2 that is evolutionarily stable for the tumor cells of this secondary site. As the tumor cell’s density grows the normal cells initially can maintain their homeostatic equilibrium of x = 40. However, as time progresses (**Panel A**), the adaptive landscape becomes depressed as the tumor cells evolve (red line in **Panel A**) and their cell density increases (**Panel B**). For a successful metastasis, the recipient organ must offer a similar environment to the one for which the tumor cells have adapted. Yet, the metastasis will initially grow very slowly with little apparent change in the normal cell density because the normal cells initially inhibit and retard the evolutionary and ecological progression of the metastasis. But, once the tumor cells have converged on the strategy required for success in the novel organ, metastatic growth and evolution will accelerate

Finally, consider a metastatic site that is ‘somewhat’ different from the primary tumor. In this case, a metastasis is limited by dispersal, establishment and diminished ecological potential. As demonstrated in Fig. 3, the cancer cell arrives at neither a fitness maximum nor at negative fitness. In this setting, the cancer cell can undergo limited proliferation—effectively forming a micro-metastasis. However, given time and opportunity this population may evolve to the fitness maximum permitting unconstrained proliferation with formation of a clinical metastasis. However, as shown, the metastatic population will, over time, evolutionarily diverge from the primary population. Furthermore, note that any tumor cell (regardless of the primary site) will find itself on and evolve in response to the novel adaptive landscape offered by the secondary tissue. In other words, site-specific convergent evolution should occur so that, e.g. a common liver-adapted cancer phenotype will tend to emerge regardless of the site of the original cancer.

### Treatment of primary cancer

**Figure 4.**
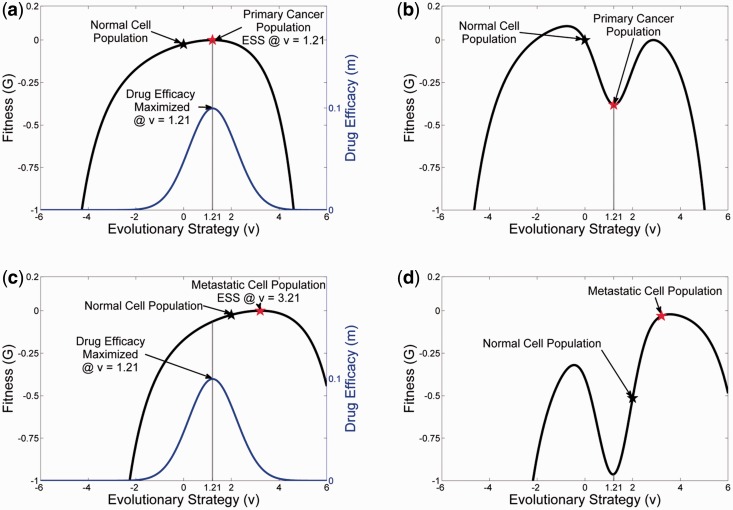
The effect of targeted therapy on the primary tumor and the secondary tumor. **Panel A** shows the adaptive landscape of the primary tumor from Fig. 1 at time = 250. The tumor cells possess a strategy of *u* = 1.2 while the normal cells have the original strategy of *u* = 0. The simulated targeted therapy (blue) treats the cancer phenotype of the primary tumor by having a maximum cytotoxic effect on cells with *u* = 1.2. **Panel B** shows how therapy with this drug changes the landscape. The fitness of the primary tumor phenotype drops below zero while that of the normal cells drops slightly. The primary tumor cells will either go extinct or over time evolve a resistant phenotype of *u* = 2.86 (the subsequent ecological and evolutionary dynamics of resistance are shown in Fig. 5). **Panel C** illustrates how the targeted therapy for the primary tumor is ineffective in the secondary tumor where *u* = 2 and *u* = 3.21 for the normal and tumor cells, respectively (evaluated at time = 250 from Fig. 3). In fact the effect of the treatment on the adaptive landscape of the secondary tumor actually favors the tumor cells over the normal cells (**Panel D**). Figure 6 shows the ecological and evolutionary dynamics caused by this therapy in the secondary tumor

Now consider an ‘ideal’ or targeted drug for some primary cancer. An ideal drug would be one that targets the specific strategies of that tumor population. Such a drug is designed as a ‘u-specific’ treatment. Here, e.g. we ‘design’ a targeted therapy with the effect greatest at u= 1.21 (Fig. 4) where the tumor population has found its evolutionarily stable strategy. Because the drug is highly targeted, its effectiveness declines in a Gaussian manner away from the specified u. For example, an anti-estrogen drug will be most effective in cancer cells with high levels of ER expression but less so in cells with no or lower ER expression [31, 32]. The rate at which the effect falls off is defined by $\sigma_{m}$. For this example we have set the drug’s efficacy to $m_{\text{max}}=0.01$ and $\sigma_{m}=1$.

(6) $$\mu =\;m_{max}*\text{exp}\left(\frac{-{\left(v-\;1.21\right)}^{2}}{\sigma_{m}}\right)$$

To apply this ideal drug to the primary tumor population a density dependent death-rate term is added to the G-function using the u -specific μ as the rate coefficient.

(7) $$G\left(v,\;u,\;x\right)=r\left(1-\;\frac{1}{K\left(v\right)}\text{n}\underset{j}{\sum }a_{j}\left(v,\;u_{j}\right)\text{n}x_{j}\right)-\mu x$$

**Figure 5.**
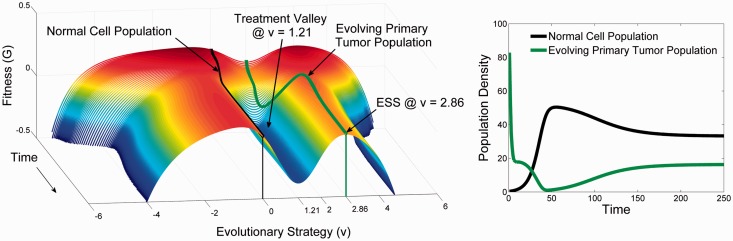
The evolutionary (**Panel A**) and ecological dynamics (**Panel B**) in the primary tumor of the therapy shown in Fig. 4 that targets cells with a phenotype of *u* = 1.2. Initially, tumor cells virtually disappear, but surviving cells at first slowly and then more rapidly evolve to a new ESS of *u* = 2.86. Despite evolving resistance, the tumor cells are held in check as the normal cells can survive, coexist and repopulate the primary site. Although this does not show a clinical cure at the primary site, this is a successful remission

The initial effect (Fig. 5) of the drug is a large reduction in the cancer population density. The tumor population then stays in this state, seemingly unchanging, simulating a prolonged, significant response (a complete response if the population is below the detection threshold or partial response if it remains detectable). Because the remaining tumor population still has the capability to adapt, it will eventually generate one or more new phenotypes that are a little to the left or to the right of the ‘ideal’ drug. This results in a very rapid climb up the landscape and then proliferation of a resistant phenotype with clinical progression.

### Targeted therapy for metastases

**Figure 6.**
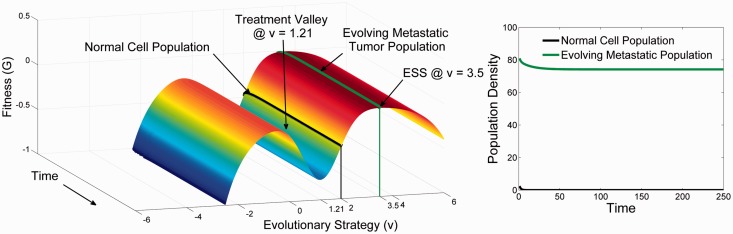
The evolutionary (**Panel A**) and ecological dynamics (**Panel B**) in the secondary tumor of the therapy shown in Fig. 4 that targets cells with a phenotype of *u* = 1.2. The divergence of the secondary tumor cells from a strategy of *u* = 1.2–3.21 means that not only is the therapy ineffective, it is counter-productive as normal cells experience much greater toxicity than the tumor cells. There is a very small decline in the tumor cells as they evolve slightly towards a new ESS of *u* = 3.5

As shown in Fig. 6, the tumor response to targeted therapy is dependent on the evolutionary dynamics at the distant site. If little or no adaptation was necessary to permit growth of a metastasis, the response will be identical to that of the primary tumor. However, if cellular adaptation to the distant site required significant evolution, the responses will diverge, and treatment will be ineffective, or worse it may hasten the evolution of more aggressive tumor cells within the recipient tissue.

## DISCUSSION

Here, we investigate the interactions of circulating cancer cells with the adaptive landscapes of a distant site using evolutionary models that explicitly incorporate the characteristics of the tumor cells with tissue-specific variations in the normal microenvironment and the interactions of these populations *in vivo*. These types of models have been extensively explored in examining invasive species in nature [33–35], which form an interesting analogy to the metastatic process.

We find the initiating tumor cell will generally arrive with a phenotype corresponding to a fitness maximum at the primary site. However, in the metastatic site the tumor cells encounter an entirely foreign landscape resulting in the potential for both divergent and convergent evolutionary dynamics.

## CONCLUSIONS AND IMPLICATIONS

Our results have several potential clinical implications. First, we demonstrate that metastatic lesions will phenotypically and genotypically diverge from the primary cancer. This is not surprising and such variations between primary and metastatic cells have been widely reported [36]. However, we also demonstrate that there will be convergence of metastatic cells within the same organ. That is, formation of metastatic tumors in any given organ will require similar strategies to successfully interact with the local mesenchyme, signaling pathways, etc. Thus, all metastases in, e.g. the liver must evolve similar ‘liver-adapted’ strategies. Thus, metastases to one organ will tend to converge on specific, organ-specific phenotypic adaptation that will result in a degree of similarity. This convergence has been observed experimentally. For example, Park *et al.* [37], using microarray to examine transcriptomes demonstrated a wide range of human cancers in mouse brains resulted in consistent re-programming of the cells to gain neuronal characteristics regardless of the organ of origin. Similarly, Chen *et al.* [38] found consistent phenotypic transitions to glucose-independent metabolism in a wide range of breast cancer metastases to the brain.

Our results suggest that current interest in identifying general molecular signatures for metastases [39, 40, 41] may be hampered by intra- and inter-tumoral heterogeneity. Within the same patient there may be different signatures for different target organs. This is because we find that the cells most likely to successfully metastasize are frequently a small minority of the cells in the primary tumor. This subset of cells may have evolved strategies in the primary tumor that make them organ-specific in their capacity to metastasize. This prediction of site-specific molecular signatures is consistent with experimental observation of a subset of genes that both enhance tumorigenicity in the primary site and augment metastatic growth in a specific distant organ [42]. That is, cellular properties that confer high probability of successful colonization at each metastatic site will probably vary depending on the specific site. In general this may limit the clinical utility of any gene signature for metastases.

Finally, we demonstrate that the predicted divergence of the tumor phenotypes in metastases from the primary population suggests that the same treatment strategy may not be equally effective in metastases that have formed in different sites or at different times. In contrast, the predicted convergence of phenotypes within organ adaptive landscapes suggests organs specific therapies may be necessary. That is, it may be necessary, e.g. to use similar treatment strategies and even targeted agents for all metastases in the liver regardless of the primary site. For example, breast cancer metastases to the brain have been found to consistently express neuronal and neuro-developmental genes [37]. Our models predict that this adaptive strategy will be broadly observed in all metastases to the brain regardless of the organ of origin. Thus, we predict that targeting specific neuronal or neuro-developmental genes could be an effective general treatment or preventative for brain metastases from many different primary sites.
